# Editorial: Crop Yield and Quality Response to the Interaction Between Environment and Genetic Factors

**DOI:** 10.3389/fgene.2022.823279

**Published:** 2022-03-18

**Authors:** Shuijin Hua, Maximiller Dal-Bianco, Zhong-Hua Chen

**Affiliations:** ^1^ Institute of Crops and Nuclear Technology Utilization, Zhejiang Academy of Agricultural Sciences, Hangzhou, China; ^2^ Laboratório de Bioquímica Genética de Plantas, BIOAGRO and Departamento de Bioquímica e Biologia Molecular, Universidade Federal de Viçosa, Vicosa, Brazil; ^3^ School of Science and Health, Western Sydney University, Penrith, NSW, Australia; ^4^ Hawkesbury Institute for the Environment, Western Sydney University, Penrith, NSW, Australia

**Keywords:** yield, quality, environment, genetic, crop

Crop yield and quality are important traits with many components. Crop yield is significantly correlated with flowering time or heading, plant height, branching or tillering, grain number, and grain weight, whereas quality is a result of metabolic flux attributed to compound biosynthesis and metabolism (Gijzen et al., 1994; Clerget et al., 2016). Although crop yield and quality are mainly controlled by genetics, the influence of the environment (including both biotic and abiotic factors) is increasingly recognized. In order to adapt to stress, the recovery of defense genes from exotic or wild relatives and re-modeling of the regulatory system may reduce damage to crops, which lack the option of physically escaping harsh environments like animals (Yu et al., 2021).

Several studies have been conducted on the genetic control and identification of important genes in crop yield and quality traits (Yin et al., 2002; Nakata et al., 2017), with a major focus on a single or limited environment(s) or gene(s). However, the effectiveness of the gene expression can differ in various environments. Therefore, it is of value to further explore the genetic and molecular mechanisms underlying the interaction between the environment and genetic factors to improve crop yield and quality.

In this Research Topic, we collected investigations on regulatory mechanisms underlying interactions between the environment and genetic factors and their effects on crop yield and quality. The Topic includes four research papers and one review.

Because of the complexities of the environment, which include climate, soil condition, and crop management practices, one of the most difficult aspects of predicting crop yield is applying appropriate models including climatic and phenotypic data and molecular markers (Kadam et al., 2016). Climatic and phenotypic data can be easily recorded with automatic instruments; however, they often fluctuate significantly. Genomic data can include QTLs, candidate genes, and molecular markers, such as SNPs, and are becoming easier and less costly to obtain. For example, Razzaq et al. reviewed candidate genes controlling fiber quality in sub-genomes A and D of cotton (*Gossypium hirsutum*), finding that chromosome 7 carries abundant SNPs for stable and potent QTLs for fiber quality.

Jarquin et al. applied five models with climatic information to study the genomic prediction efficiency of corn yield. The exclusion of the environment led to greater error variation, implicating the importance of environmental effects. Improvement in the prediction ability has been observed when the environmental data is included, as compared to prediction ability with only parental data (general or specific combining ability). The prediction ability also depends on the nature of the data provided. Normally, prediction models that used tested genotypes in the observed environment were the most accurate, whereas models based on both untested genotypes and unobserved environments were the least accurate. In partial summary, including environmental factors improves the genomic prediction of crop yield.

Regardless of the efficacy of genomic prediction, breeders and researchers should further elucidate the effects of different types of environments on crop yield and quality. One of the problems in obtaining high yields is soil stress, such as salinity. Crops have very strong plasticity and multiple pathways to avoid salt stress (Zörb et al., 2019). When plants are subjected to salt stress, they usually accumulate excessive Na^+^. There is a type of NXH (sodium/hydrogen antiporter) gene family that can regulate the equilibrium of sodium and hydrogen ions. Fu et al. found that NXH genes are a large family in the four cotton sub-species. Interestingly, these genes can be divided into three types: endosome-, plasma membrane-, and vacuole-type. The respective types may be responsible for the different functions, such as the control of gene behavior, transport of sodium and hydrogen exchange, or accumulation.

In addition to modulation of sodium/hydrogen ions in cells to improve crop salt adaptability, Ca^2+^ ions, as a means of signal transduction, play a significant role in mediating salt stress (Henriksson and Henriksson, 2005). Calmodulin-binding transcription activator (CAMTA) is a class of transcription factor that is highly related to the content of calmodulin, which in turn is responsive to Ca^2+^ ion content (Snedden and Fromm, 2001). Yuan et al. identified three subfamilies of *CAMTAs* in both *Cucurbita maxima* and *C. moschata*, although they have different numbers of activators. Cis-acting element analysis showed the structure of the genes and indicated that most of them had a putative function in salt resistance.

Drought stress is another adverse and widespread environmental response that severely affects crop yield (Mendanha et al., 2020). Although mechanisms of resistance to drought stress have been widely studied, many still remain unclear. The NAC (an acronym for the NAM, ATAF, and CUC) superfamily of transcription factors plays a vital role in the regulation of drought stress (Deng et al., 2019). Ferrira et al. investigated the function of one NAC gene in soybean (*Glycine max*), *GmNAC81*, via overexpression under drought stress. Because *GmNAC81* acts as an executioner of the cell death program, the plants had increased sensitivity to drought stress, exhibiting faster water loss in the leaves and reduced photosynthetic efficiency. Furthermore, the expression of key regulators and effectors of ABA signaling was blocked when *GmNAC81* was over-expressed. Knockout of the gene via genome editing may provide more evidence to elucidate its function.

In conclusion, precise genomic prediction using environmental, phenotypic, and genomic data is a desirable trend for breeding. The mechanism of even a single environmental stressor, such as salt or drought stress, can be modulated by different gene families, for example including NXH, CAMTA, and NAC. Further identification and evidence of the functions of these transcriptional factors are of importance.
